# All Endocarditis Is Not Infective: Libman-Sacks Endocarditis

**DOI:** 10.7759/cureus.26526

**Published:** 2022-07-03

**Authors:** Hilal Al Riyami, Niranjan Joshi, Khalfan Al Senaidi, Noof Al 'Abdul Salam, Reem Abdwani

**Affiliations:** 1 Department of Child Health, Sultan Qaboos University Hospital, Muscat, OMN; 2 Department of Child Health, Oman Medical Specialty Board, Muscat, OMN; 3 Department of Child Health, College of Medicine and Health Sciences, Muscat, OMN

**Keywords:** heart failure., endocarditis, systemic lupus erythematosus, mitral valve, libman-sacks endocarditis

## Abstract

Libman-Sacks endocarditis (LSE) is an uncommon disorder that might be confused with infective endocarditis. It is one of the systemic lupus erythematosus (SLE) manifestations that could present with heart failure. We report a 12-year-old girl who presented with a history of shortness of breath, joint pain for four weeks, and fever for about one week. On examination, she was pale, edematous, and febrile. Her cardiac exam revealed a pan-systolic murmur of mitral regurgitation, harsh, grade 3/6 best heard at the apex. She was diagnosed with systemic lupus erythematosus with lupus nephritis and carditis. Her echocardiography revealed severe mitral regurgitation with nodular thickening of the valve in keeping with a diagnosis of LSE. After appropriate management of her underlying disorder using immunosuppressive, we saw a dramatic clinical improvement and her heart failure symptoms resolved. This case proves that SLE can have significant cardiac involvement and a proper evaluation would help in overall management and prognosis.

## Introduction

Libman-Sacks endocarditis (LSE) is a rare cardiac manifestation of systemic lupus erythematosus and it could easily be confused with infective endocarditis, especially in children with a normal heart structure [1-4]. The manifestations of both conditions can overlap, and misdiagnosis of LSE can lead to severe cardiac and systemic complications [3]. We reported this case as her symptoms were mimicking those of infective endocarditis and showed dramatic improvement after immunosuppressive medication. To the best of our knowledge, no previous pediatric case was reported with such presentation and a dramatic improvement in cardiac symptoms after systemic lupus erythematosus (SLE) management.

## Case presentation

A 12-year-old girl, previously well, presented with a one-month history of shortness of breath with activities and migratory multiple small and large joints pain. This was associated with one-week intermittent fever, lower limb edema, and generalized weakness. She reported having a low mood and easy fatigability. Her past medical history and birth history were unremarkable with no history of cardiac diseases.

On assessment, she was pale, with puffy eyes and bilateral lower limbs edema. There was no lymphadenopathy. She was hemodynamically stable except for a temperature of 39°C and her blood pressure of 119/78 (at 90th centile). Chest examination was normal and cardiac examinations revealed normal S1 S2 with a loud grade 4/6 pan-systolic murmur best heard at the apex with a gallop rhythm radiating all over her precordium. Laboratory investigations revealed hypochromic microcytic anemia with thrombocytopenia and acute kidney injury (Table 1). Cardiac enzymes were very high, and she had low C3 complement and C4 complement and positive anti-dsDNA (Table 1).

**Table 1:** Laboratory results at the time of presentation.

Labs	Results		Reference range
Hemoglobin	8.8 g/dL		11.5-15.5 g/dL
Haematocrit	0.28 L/L		0.350-0.450 L/L
Platelet	81 × 10^9^/L		150-450 × 10^9^/L
White Cell Count	12 × 10^9^/L		4.5-14.5 × 10^9^/L
Urea	7 mmol/L		2.8-8.1 mmol/L
Creatinine	84 μmol/L		39-60 μmol/L
Troponin T	162 ng/L		<14 ng/L
C-Reactive Protein	71 mg/L		0-5 mg/L
Antinuclear antibody	Positive >640		0-<4
Anti-double-stranded DNA	>600 IU/ml		0-9 IU/ml
C3 complement	0.54 g/dL		0.9–1.8 g/dL
C4 complement	0.02 g/L		0.1–0.4 g/dL

**Figure 1:**
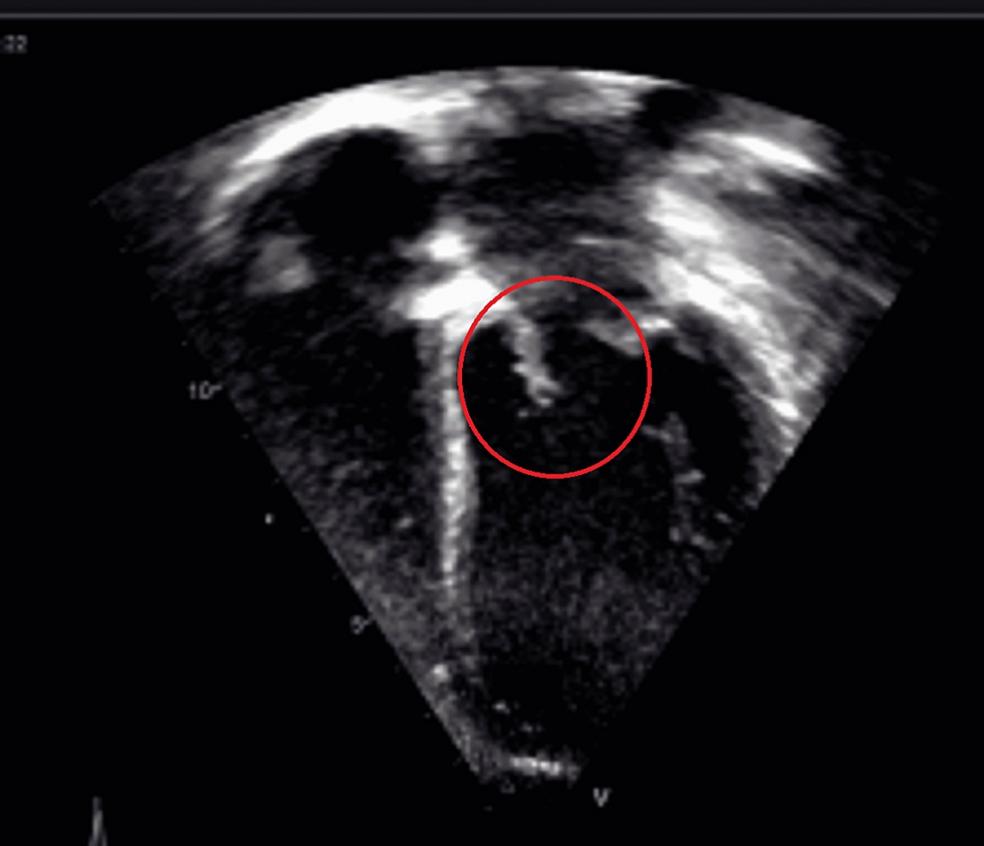
Echocardiography (Apical four-chamber view showing thick anterior mitral leaflet)

**Figure 2:**
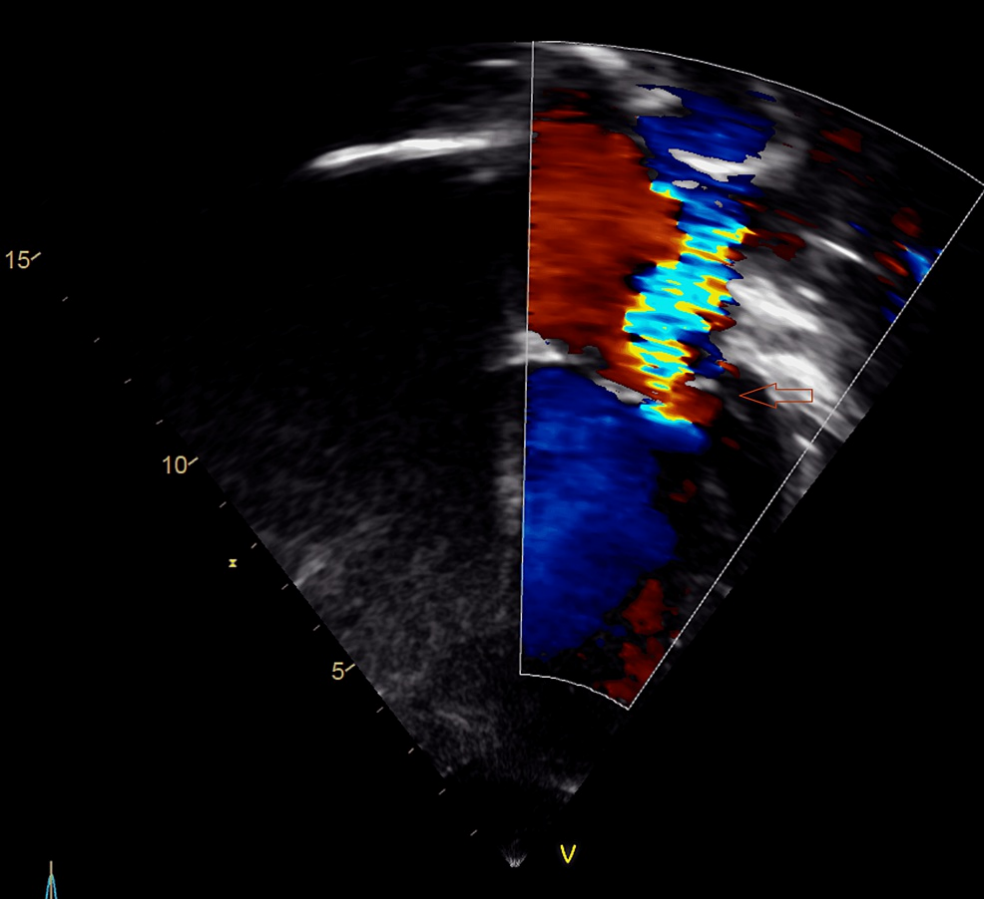
Echocardiography (Apical four-chamber view showing severe mitral regurgitation)

The blood culture and urine culture were negative. A renal biopsy showed: focal proliferative lupus nephritis class III with membranous lupus nephritis class V (ISN/RBS). We entertained the possible differential diagnosis of mixed connective tissue disease, SLE, and infective endocarditis. Chest X-ray showed cardiomegaly with congested lungs and her electrocardiogram (ECG) showed normal sinus rhythm with prolonged PR interval and normal voltage. The echocardiography showed thickening of the anterior mitral leaflet with severe mitral valve regurgitation (Figures 1, 2), mildly dilated left atrium with Z score 2.5, no left ventricular dilation, normal ventricular systolic function and a small rim of pericardial effusion.

**Figure 3:**
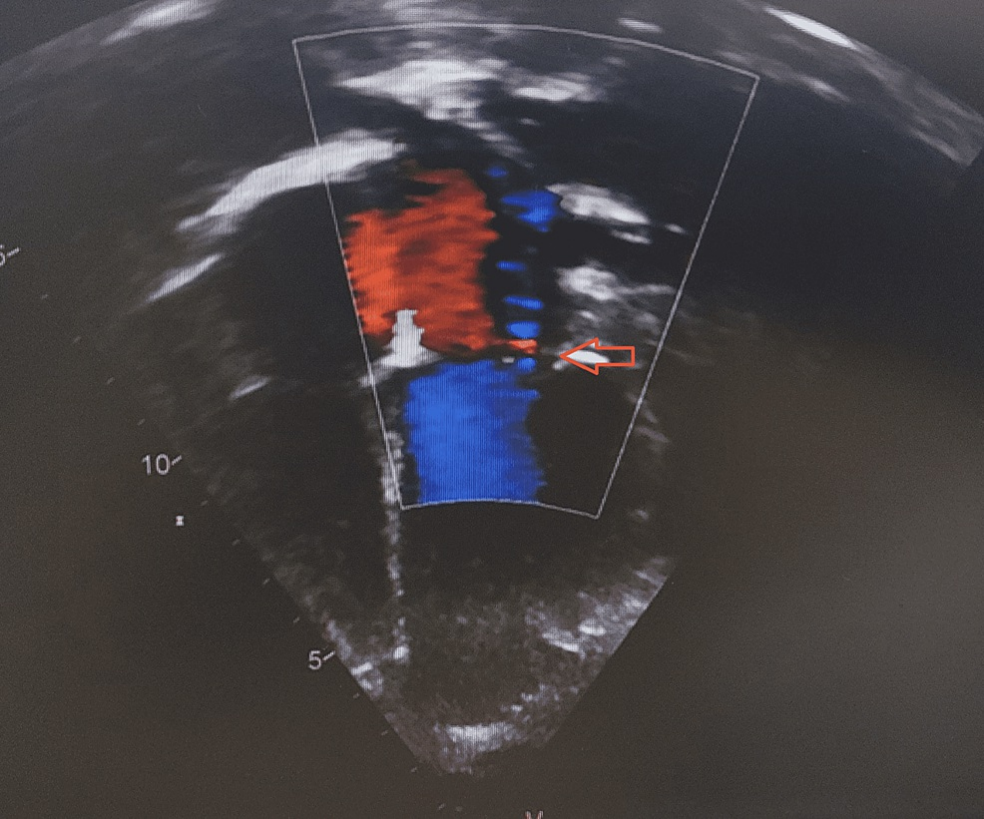
Echocardiography (Apical four-chamber view showing mild Mitral regurgitation)

She was managed as systemic lupus erythematosus with nephritis and Libman-Sacks endocarditis based on her clinical and investigation finding. She was hospitalized for 20 days during which her SLE flare was treated properly with intravenous (IV) methylprednisolone and cyclophosphamide. On discharge, she was on a tapering dose of oral prednisolone for six months and monthly IV cyclophosphamide and on regular hydroxychloroquine, esomeprazole, calcium and vitamin D. The heart failure symptoms were controlled by congestive heart failure medication including furosemide, spironolactone and afterload reduction using captopril. After four months, the echocardiography showed mild mitral regurgitation and reduced thickness of the anterior leaflet of the mitral valve (Figure 3). She was clinically back to her normal life and her heart failure medication was weaned slowly. The SLE flare-up was controlled and she is still under follow-up by the Rheumatology team, Cardiology, and Nephrology team.

## Discussion

Libman-Sacks endocarditis is a distinctive heart manifestation in the presence of systemic lupus erythematosus [1,3]. However, it is rare in the pediatric age group with not enough evidence about the management [2,4]. SLE can present as pericarditis, arrhythmias, abnormal conduction, myocarditis, and increased pulmonary pressure [1,2]. Life-threatening manifestations like cardiac tamponade and cardiogenic shock could be the initial presentation [4].

Libman and Sacks described valvular disease due to SLE for the first time in 1924 [1,3,5]. LSE is sterilized vegetation that mostly affects the mitral valves and possibly the aortic valve [3,5]. It may present as infective endocarditis with symptoms of heart failure due to valvular regurgitation and thromboembolic events [6]. LSE is typically situated in the tip, middle, or the base of the posterior mitral leaflets and this can be confused with infective endocarditis vegetation [7-10].

Diagnosing LSE can be difficult in the absence of cardiac symptoms for which it is recommended to do regular echocardiography screening for SLE patients even if there are no symptoms from a cardiac viewpoint [2]. A high index of suspicion for LSE in the presence of multiorgan disease is warranted as early recognition of LSE could have an impact on its management and reduce morbidity and mortality [8]. According to pathology reports from valvular specimens, the underlying mechanism for LSE showed evidence of inflammation and cell degeneration with fibrin deposition [10-11].

Management of LSE and the usefulness of immunosuppressive for valvular regurgitation were not described in detail before in the pediatric age group but some evidence in the adult population [8,12-13]. For instance, Ishizu et al. [10] reported a dramatic improvement after using immunosuppressive medication in LSE with severe mitral regurgitation. However, there was a dramatic improvement in this case after proper SLE management and mitral regurgitation almost resolved in about four months from the initial presentation. In addition, since the surgical treatment of valvular diseases associated with LSE has a higher morbidity and mortality rate, it is worth starting medical therapy with immunosuppressive medication [1,3].

## Conclusions

Libman-Sacks endocarditis is a rare condition that can be missed easily as in this case. It can present with severe cardiac manifestation which should be recognized and differentiated from infective endocarditis as the former needs appropriate and timely immunosuppression which may worsen the latter condition. Appropriate treatment of the SLE flare-up usually decreases the severity and residual damage due to LSE and improved the long-term outcome. Furthermore, heart failure must be managed effectively and regular cardiac evaluation should be continued even with normal heart function.
